# Frequency and risk factors of low immunoglobulin levels in patients with inflammatory bowel disease

**DOI:** 10.1093/gastro/gou082

**Published:** 2015-01-30

**Authors:** Tarun Rai, Xianrui Wu, Bo Shen

**Affiliations:** Departments of General Internal Medicine and Gastroenterology/Hepatology, the Cleveland Clinic Foundation, Cleveland, OH, USA

**Keywords:** inflammatory bowel disease, immunoglobulins, biologics, Crohn’s disease, ulcerative colitis, ileal pouch

## Abstract

**Background and aims:** Inflammatory bowel diseases (IBD) are considered to be dysregulated, immune-mediated disorders; and immunosuppressive medications are the mainstay of their treatment. Clinically, we have often observed low serum immunoglobulin (Ig) levels in these patients. The aim of this study was to assess the frequency and risk factors of secondary humoral immunodeficiency in IBD patients.

**Methods:** We conducted a cross-sectional study of eligible IBD patients with Crohn’s disease (CD), ulcerative colitis (UC), indeterminate colitis (IC) or restorative proctocolectomy with ileal pouch, who having serum Ig measured. Demographic and clinical variables were measured. Univariate and multivariate analyses were performed.

**Results:** A total of 324 patients was included, with a mean age of 38.8 years and 158 (48.8%) being male. Low IgG, IgG1, IgA, and IgM were found in 22.7%, 23.4%, 7.9%, and 10.9% of patients, respectively. The shared risk factors for a low IgG or IgM level were increasing age [odds ratio (OR) = 1.13; 95% confidence interval (CI) 1.03–1.23 for low IgG level and OR = 1.33; 95% CI 1.15–1.56 for low IgM level] and hypoalbuminemia (OR = 1.83; 95% CI 1.01–3.33 for low IgG level and OR = 3.17; 95% CI 1.23–8.15 for low IgM level). In addition, thioprine use was associated with low IgA level (OR = 2.76; 95% CI 1.03–7.39). IBD disease duration was a risk factor for low IgG1 level (OR = 1.40; 95% CI 1.12–1.76). The presence of concurrent primary sclerosing cholangitis (OR = 0.064; 95% CI 0.007–0.60) and the use of biologics (OR = 0.16; 95% CI 0.033–0.79) were associated with normal IgG1 level. IgG level was lower in CD patients than that in UC/IC and ileal pouch patients (*P* = 0.042). IgG and IgA levels were elevated in patients with inflammatory conditions of the pouch (*P* = 0.01; *P* = 0.003, respectively).

**Conclusions:** Low Ig level appears to be common in IBD patients. Increasing age, disease duration and hypoalbuminemia appeared to be risk factors. The findings may provide rationale for targeted therapy to boost humoral immunity in selected patients with IBD.

## Introduction

Since the initial description of ileitis in 1913, multiple theories have been proposed to understand the etiology and pathogenesis of inflammatory bowel disease (IBD), including infection [1], psychosomatic dysfunction [2], dietary allergens and sulfur compounds [3], the use of newer toothpastes [4] and altered diet hygiene [5]. The current theory maintains that the pathogenesis of IBD is associated with an abnormal immune response to altered gut microbiota in genetically susceptible individuals. The objective of medical management has been immune system suppression or modulation, to control inflammation and disease activity. Commonly used agents include corticosteroids, immunomodulators, and anti-tumor necrosis factor (TNF) biologics, but we still see a great number of patients undergoing surgery for failed medical treatment and the frequency of surgery over long term has not changed significantly in the current ‘biological era' [6, 7].

Reported findings in the study of immunocompetence in IBD patients are highly variable. Studies of the serum IgG subclass revealed that there were elevated IgG1 and IgG3 levels in ulcerative colitis (UC) and increased IgG2 and IgG4 evels in CD patients [8–10]. A decrease in serum IgG or IgM levels following treatment with sulfasalazine or prednisone has been reported in clinical trials [11, 12]. On the other hand, an increase in serum IgM level was noted after abdominal surgery in IBD patients [13, 14]. No fixed pattern of Ig levels associated with disease activity was noted [15–17]. Since the clinical utility of measuring Ig level in IBD patients was not proven, it is not common to measure the level in routine practice. In the background of those conflicting results, we have noted that, in our clinical experience, low Ig levels are common in IBD patients and some patients with refractory IBD responded to intravenous immunoglobulin (IVIG) therapy [18–21]. Therefore, we initiated this cross-sectional study to assess the frequency of low serum Ig in IBD patients and risk factors associated with Ig deficiency.

## Patients and methods

### Patients

The cross-sectional study was approved by the Cleveland Clinic Institutional Review Board. Patients were identified by the Department of E-research after scanning charts of the Cleveland Clinic Health System patients, both at the Cleveland and Florida campuses, maintained in an EPIC® database.

We collected this information from April 2002 to March 2012. All the patients having a diagnosis of CD, UC, indeterminate colitis (IC) or restorative proctocolectomy with ileal pouches (for IBD indication) and whose IgG, IgG1, IgA and IgM levels were measured were searched. The disease diagnosis and laboratory tests were searched through ICD-9 codes and the laboratory codes, respectively. A total number of 1528 patients was initially identified after matching the codes. After reviewing the charts, 324 patients were included who satisfied inclusion criteria. Rest 1024 were excluded. The inclusion criteria were (i) confirmed diagnosis of IBD and (ii) measured serum IgG, IgG1, IgM or IgA levels. The exclusion criteria were patients with (i) primary immune deficiency disorders and (ii) secondary immune deficiency due to HIV infection.

### Measurement of serum immunoglobulin

Patients were classified into two categories: (i) low or normal or (ii) high Ig status, based on our laboratory reference standard for individual Igs. The cut-off levels for IgG, IgG1, IgG2, IgG3, IgG4, IgA, and IgM deficiency were any value lower than 717 mg/dL, 456 mg/dL, 125 mg/dL, 25 mg/dL, 11 mg/dL, 78 mg/dL, and 53 mg/dL, respectively. All patients included in the study had either IgG or IgG1 measured at the time of inclusion. The majority of patients had immunoglobulin GAM (IgG, IgA or IgM) or IgG subclass measured at the time of initial presentation.

### Diagnostic criteria

The diagnoses of CD and UC were made, based on a combined assessment of clinical, endoscopic, radiographic, and histological features. The disease location was retrospectively verified, based on the Montreal Classification [22]. Disease severities for the CD and UC patients were assessed with the Harvey-Bradshaw index [23] and modified Mayo Score [24], respectively. CD phenotype was based on the Vienna Classification [25]. For analysis, the disease activity in UC/CD patients was further divided into two simple groups: (i) remission to mild activity and (ii) moderate-to-severe activity. Pouch patients were divided into three categories: (i) no inflammation, (ii) inflammatory conditions of the pouch (pouchitis, CD of the pouch, and cuffitis) and (iii) surgical procedure-associated complications (such as anastomotic sinus, afferent and efferent limb syndromes, and pouch prolapse).

### Demographic and clinical variables

Basic demographics, social and family histories, ages at the disease diagnosis, ages at the time of Ig measurement, disease activity and severity, concurrent medications, laboratory tests including complete blood count, basic metabolic profile, and serum calcium, albumin, liver enzymes, ferritin, vitamin D, and stool *Clostridium difficile* toxins were documented. Only laboratory values present at the time of serum Ig measurement, or within 6 months thereafter, were included. Concurrent primary sclerosing cholangitis (PSC), history of colon cancer, bowel surgeries and complications for each disease diagnosis and IBD-related hospital admissions were recorded for the period two years before and after the Ig levels were taken. Data were entered into an Excel™ spreadsheet and saved in a dedicated password-protected memory disk provided by the Cleveland Clinic. The utmost care was taken to protect the confidential patient information, with access allowed only to the persons involved in the research.

### Outcome measurements

The primary outcome was the assessment of frequency and risk factors of low Ig level in this cohort.

### Statistical analysis

Descriptive statistics were computed for all variables. These included means and standard deviations (SD) or medians and interquartile ranges (IQR) for continuous factors, and frequencies for categorical factors. Patients with and without immunoglobulin deficiency acted as cases and controls, respectively. Using univariate analysis, a total of 34 demographic and clinical variables was analysed for risk factors for each Ig deficiency separately. A multivariate logistic regression analysis was constructed using the forward stepwise method with an entry criterion of *P* < 0.05 and a removal criterion of *P* > 0.10. The variable “Use of biologics” was forced into the final model. Ig levels were compared in CD, or UC and pouch patients, using the Kruskal-Wallis test. The correlation of immunoglobulin level with disease activity in UC and CD and pouch patients was measured by Spearman rank correlation analysis. All statistical analyses were performed using SPSS software version 16 (SPSS, Chicago, IL). A *P*-value below 0.05 was considered statistically significant.

## Results

### Demographic and clinical characteristics

Out of 1528 who were screened, a total of 324 patients met the inclusion criteria and were studied. The study cohort included 158 (48.8%) males, 185 (57.1%) with CD, 41 (12.7%) with UC, and 98 (30.2%) with ileal pouches. The mean age of the patient cohort was 38.8 ± 17.8 years. The mean age at diagnosis of IBD was 27 ± 15.5 years. The median duration from IBD diagnosis to Ig measurement was 8.3 years [interquartile range (IQR) 1.7–19.5]. Of the total number, 31% and 39% of the patients were using 5-aminosalicylates (5-ASA) or corticosteroids, respectively, whereas 17.4% and 15.8% used 6-mecaptopurine (6-MP)/azathioprine or anti-TNF biologics, respectively. The demographic and clinical characteristics are summarized in Table 1.

**Table 1.** Demographic and clinical characteristics

Characteristic	All cases (*n* = 324)
Age at diagnosis of IBD, years	27.0 ± 15.5
Age at Ig test, years	38.8 ± 17.8
Duration from IBD to Ig test, years (interquartile range)	8.3 (1.7–19.5)
Male gender, *n* (%)	158 (48.8%)
Caucasian patients, *n* (%)	275 (84.9%)
Body mass index, kg/m^2^	25.4 ± 6.0
Smoking status, *n* (%)	
Never	215 (67.0%)
Current and past smoker	106 (33.0%)
Alcohol, *n* (%)	
Never	214 (66.5%)
Current or past heavy use of alcohol	108 (33.5%)
Family history of IBD, *n* (%)	73 (22.7%)
Family history of colorectal cancer, *n* (%)	35 (10.9%)
History of IBD related bowel surgery, *n* (%)	223 (68.8%)
Type of IBD, *n* (%)	
CD	194 (59.9%)
UC or IC	130 (40.1%)
Ileal pouch, *n* (%)	98 (30.2%)
Toxic or fulminant colitis, *n* (%)	29 (9.0%)
Fistulizing or stricturing CD phenotype, *n* (%)	116 (35.8%)
Primary sclerosing cholangitis, *n* (%)	38 (11.8%)
IBD-related hospitalization in the last two years, *n* (%)	165 (53.1%)
Medications, *n* (%)	
5-aminosalicylic acids	100 (31.0%)
Corticosteroids	126 (39.0%)
6-mercaptopurine/azathioprine	56 (17.4%)
Anti-TNF biologics	51 (15.8%)
Methotrexate	15 (4.6%)
Antibiotics	129 (39.8%)
Laboratory tests, *n* (%)	
Anemia	197 (61.2%)
Thrombocytosis	70 (21.7%)
Leukocytosis	56 (17.4%)
Hypoalbuminemia	102 (32.1%)
Elevated alkaline phosphatase	57 (18.0%)
Elevated aspartate aminotransferase	48 (15.2%)
Elevated alanine aminotransferase	50 (15.8%)
Increased total bilirubin	26 (8.2%)
Hypocalcemia	63 (19.9%)
Low vitamin D	123 (74.5%)
Low ferritin level	29 (18.2%)
*C. difficile-*positive	6 (2.7%)

CD = Crohn’s disease; IC = indeterminate colitis; IBD = inflammatory bowel disease; TNF = tumor necrosis factor; UC = ulcerative colitis.

### Frequency of low immunoglobulin level

Decreased IgG level was found in 65 patients (22.7%), low IgG1 level in 29 (23.4%), low IgA level in 21 (7.9%), and low IgM level in 24 (10.9%). Demographic features, smoking and alcohol history, age of IBD diagnosis, age of serum Ig level measurement, disease diagnosis, medication use and laboratory tests were equitably distributed among patients of each low Ig groups (Supplementary Tables 1–4).

### Risk factors for low immunoglobulin levels

On univariate analysis for IgG deficiency; age at the time of Ig measurement, duration of IBD diagnosis, CD diagnosis, fistulizing or stricturing phenotype of CD, hypoalbuminemia and hypocalcemia were found to be significantly associated with low Ig levels. For patients with a low IgG1, duration of IBD diagnosis, smoking, the absence of PSC, anemia and hypoalbuminemia were significant risk factors. For those with a low IgA, non-smoking, immunomodulator use and thrombocytosis were significant risk factors. Besides age at IBD diagnosis, other risk factors for a low IgM were older age at Ig measurement, duration of IBD diagnosis, high body mass index (BMI), no family history of IBD, corticosteroid use, hypoalbuminemia, and hypocalcemia. (Table 2).

**Table 2.** *P-*values of univariate analysis of risk factors for low Ig level

Characteristic	Low IgG level (*n* = 65)	Low IgG1 level (*n* = 29)	Low IgA level (*n* = 21)	Low IgM level (*n* = 24)
Age at diagnosis of IBD, years	0.181	0.229	0.868	0.001
Age at Ig test, years	0.001	0.305	0.612	<0.001
Duration from IBD to Ig test, years	0.004	0.011	0.372	0.006
Male gender	0.162	0.445	0.378	0.503
Caucasian patients	0.054	1.0	1.0	0.774
Body mass index, kg/m^2^	0.517	0.587	0.115	0.037
Current and past smoker	0.162	0.010	0.022	0.671
Regular alcohol use	0.423	0.101	0.056	0.659
Family history of IBD	0.682	0.815	0.402	0.025
Family history of colorectal cancer	0.532	0.189	0.256	1.0
History of IBD-related bowel surgery	0.062	0.278	0.538	0.173
Ileal pouch construction	0.052	0.138	0.607	0.159
Type of IBD (CD *vs.* UC or IC)	0.042	0.118	0.752	0.123
Toxic or fulminant colitis	0.817	1.0	0.233	0.495
Fistulizing or stricturing CD phenotype	0.001	0.903	0.101	0.784
Primary sclerosing cholangitis	0.054	0.010	0.704	0.641
IBD-related hospitalization in last two years	0.574	0.166	0.270	0.583
Medications				
5-aminosalicylic acids	0.595	0.303	0.520	0.359
Corticosteroids	0.118	0.464	0.958	0.014
6-mercaptopurine/azathioprine	0.636	0.559	0.035	1.0
Anti-TNF biologics	0.180	0.161	1.0	1.0
Methotrexate	0.204	1.0	0.608	1.0
Antibiotics	0.488	0.989	0.588	0.734
Laboratory tests				
Anemia	0.124	0.001	0.994	0.191
Thrombocytosis	0.344	0.472	0.033	0.071
Leukocytosis	0.830	0.635	0.392	1.0
Hypoalbuminemia	0.008	0.004	0.620	0.004
Elevated alkaline phosphatase	0.843	0.670	0.394	0.393
Elevated aspartate aminotransferase	0.524	0.779	0.755	0.722
Elevated alanine aminotransferase	0.264	0.217	0.748	0.556
Increased total bilirubin	0.792	1.0	0.089	0.623
Hypocalcemia	0.009	0.120	0.581	0.006
Low vitamin D	0.688	0.532	0.295	1.0
Low ferritin	0.730	1.0	0.690	0.741
*C. difficile-*positive	0.324	0.101	0.662	1.0

CD = Crohn’s disease; IBD = inflammatory bowel disease; IC = indeterminate colitis; TNF = tumor necrosis factor; UC = ulcerative colitis.

On multivariate analysis, common risk factors for a low IgG or IgM level were increasing age [odds ratio (OR) = 1.13; 95% confidence interval (CI) 1.03–1.23; *P* = 0.006 for a low IgG and OR = 1.33; 95% CI 1.15–1.56; *P* < 0.001 for a low IgM] and hypoalbuminemia (OR = 1.83; 95% CI 1.01–3.33; *P* = 0.046 for a low IgG and OR = 3.17; 95% CI 1.23–8.15; *P* = 0.017 for a low IgM). Immunomodulator use was associated with IgA deficiency (OR = 2.76; 95% CI 1.03–7.39; *P* = 0.044). Increased disease duration of IBD was a risk factor for a low IgG1 (OR = 1.40; 95% CI 1.12–1.76; *P* = 0.004). On the other hand, the presence of PSC (OR = 0.064; 95% CI 0.007–0.60; *P* = 0.016) and the use of anti-TNF biologics (OR = 0.16; 95% CI 0.033–0.79; *P* = 0.024) were associated with normal IgG1 level. The use of anti-TNF biologics was not associated with a low level of any other Ig deficiency (Table 3).

**Table 3.** Multivariate analysis of risk factors associated with low Ig level

Characteristic	Odds ratio	95% confidence interval	*P*-value
**Low IgG level**			
Age at Ig level, every 5-year increase	1.13	1.03–1.23	0.006
Crohn’s disease-related complications	2.48	1.38–4.49	0.003
Hypoalbuminemia	1.83	1.01–3.33	0.046
Anti-TNF biologics	0.49	0.20–1.20	0.12
**Low IgG1 level**			
Duration of IBD; every 5-year increase	1.40	1.12–1.76	0.004
Ever smoked?	2.70	0.85–8.52	0.091
Primary sclerosing cholangitis	0.064	0.007–0.60	0.016
Anemia	9.27	2.47–34.99	0.001
Anti-TNF biologics	0.16	0.033–0.79	0.024
**Low IgA level**			
6-mercaptopurine/ azathioprine use	2.76	1.03–7.39	0.044
Thrombocytosis	2.72	1.07–6.90	0.035
Anti-TNF biologics	0.97	0.27–3.52	0.96
**Low IgM level**			
Age at Ig level; every 5-year increase	1.33	1.15–1.56	<0.001
Hypoalbuminemia	3.17	1.23–8.15	0.017
Anti-TNF biologics	1.11	0.32–3.78	0.87

IBD = inflammatory bowel disease; TNF = tumor necrosis factor.

### Association of immunoglobulin levels with disease diagnosis and activity

**Figure 1.**
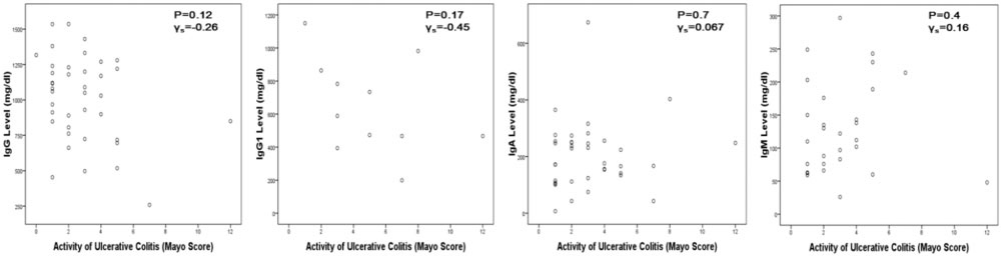
Correlation of Ig level and disease activity in UC patients. (a) IgG; (b) IgG1; (c) IgA; (d) IgM.

**Figure 2.**
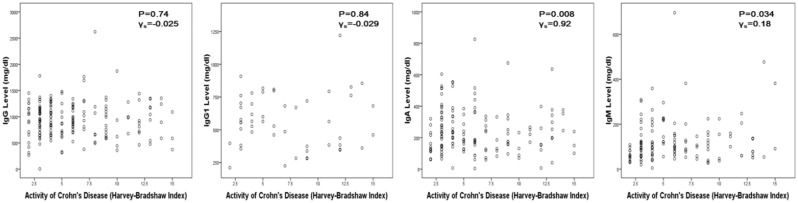
Correlation of Ig level and disease activity in CD patients. (a) IgG; (b) IgG1; (c) IgA; (d) IgM.

IgG level was lower in CD patients than in UC or IC and ileal pouch patients (*P* = 0.042). However, no significant difference was identified in IgG1, IgA or IgM levels among the three groups (all *P* > 0.05) (Table 4). IgG and IgA levels in pouch patients with inflammation were higher than the pouch patients without complications (*P* = 0.01 and *P* = 0.003, respectively). For other groups, there was no correlation between Ig levels and disease activity (Table 5). This was further illustrated by Figures 1 and 2.

**Table 4.** Comparison of immunoglobulin levels in various forms of inflammatory bowel disease

Ig levels	All patients (*n* = 324)	CD (*n* = 185)	UC or IC (*n* = 41)	Ileal pouch (*n* = 98)	*P-*value
IgG	980.5 (747.3–1200.0)	940.5 (697.0–1170.0)	1060.0 (806.0–1230.0)	1040.0 (819.5–1290.0)	***0.042***
IgG1	577.5 (457.3–760.3)	556.0 (384.0–720.0)	589.0 (467.0–864.0)	634.0 (485.3–765.8)	0.25
IgA	223.0 (142.0–312.5)	211.5 (140.5–325.3)	176.0 (119.5–252.5)	229.5 (166.0–331.0)	0.062
IgM	107.5 (64.5–164.8)	99.5 (60.8–152.3)	112.0 (71.0–182.5)	115.0 (85.0–203.0)	0.099

Ig levels were measured in mg/dL and described as medians and interquartile ranges.

CD = Crohn’s disease; IC = indeterminate colitis; UC = ulcerative colitis.

**Table 5.** Immunoglobulin levels and IBD disease activity

Ig levels	Crohn’s disease			Ulcerative/indeterminate colitis			Ileal pouch			
	Remission/mild (*n* = 127)	Moderate/severe (*n* = 55)	*P*	Remission/mild (*n* = 28)	Moderate/severe (*n* = 13)	*P*	Normal (*n* = 26)	Inflammation (*n* = 38)	Surgical complication (*n* = 29)	*P*
IgG	943 (729–1164)	923 (612–1185)	0.37	1085 (858–1237)	899 (696–1220)	0.22	893 (668–1185)	1180 (910–2100)	1035 (825–1212.5)	0.028
IgG1	565 (484–732)	437 (348–741)	0.22	783 (491–1007)	470 (400–796)	0.27	506 (332–691)	640 (495–897)	658 (480–749)	0.13
IgA	218 (137–334)	200 (131–298)	0.56	231 (110–264)	166 (144–242)	0.66	190 (115–286)	286 (201–432)	245 (163–322)	0.1
IgM	97 (61–149)	102 (58–153)	0.84	97 (66–150)	140 (91–218)	0.27	109 (62–186)	113 (93–182)	119 (78–254)	0.75

Ig levels were measured in mg/dL and described as medians and interquartile ranges.

Disease activity data for three Crohn’s disease patients and five ileal pouch patients were missing.

## Discussion

Our study showed that low Ig levels were common in IBD patients. The decrease in each Ig class was associated with different risk factors. Increasing age and the presence of low serum albumin were shared common risk factors for a low serum IgG or IgM level. A long duration of IBD was a risk factor for a low IgG1 level. Mean IgG level was low in CD, as compared with other forms of IBD. Use of thioprine was associated with low IgA level. On the other hand, anti-TNF biologics were found to be associated with normal IgG1 and trended towards a normal IgG level.

Secondary immune deficiency is more common than primary immune deficiency in clinical practice. Igs are secreted by plasma cells as a complex interplay of T and B cells, antigens and antigen-presenting cells. Ig deficiency, either primary or secondary, represents a subset of a disturbed humoral immune system, due to quantitative or qualitative failure of plasma cells to secrete serum Ig. The most common causes of primary immunoglobulin deficiency are common variable immunodeficiency, selective IgA deficiency, transient hypogammaglobulinemia in infancy, IgG subclass deficiency and partial antibody deficiency with impaired polysaccharide responsiveness. Immunosuppressant drugs, such as corticosteroids [26, 27], rituximab [28–30], methotrexate [31], 6-MP/azathioprine [32] and antithymocyte globulin can cause temporary or permanent Ig deficiency. Other drugs, such as gold [33], D-penicillamine [34], sulfasalazine [11, 12] and anticonvulsants, can also decrease Ig secretion. Other factors associated with secondary Ig deficiency include nephrotic syndrome, uremia with or without dialysis, cirrhosis, diabetes, malnutrition, viral and bacterial infections [35]. With malnutrition and increasing age, Ig level could be normal or high, but antibody production in response to antigens is decreased [36, 37].

The pathogenic role of Ig in IBD patients has been studied; as early as the 1960s, it was speculated that IBD results from dysfunction in the innate immune system [38]. On the other hand, the gastrointestinal tract is commonly involved in primary immunodeficiency syndromes, such as chronic granulomatous disease and Chediak-Higashi syndrome. There has recently been renewed interest in the Ig deficiency theory, with studies showing that infusion of intravenous Ig [18–21] and granulocyte colony-stimulating factor (G-CSF) [39, 40] could induce and maintain remission in patients with medically refractory IBD. Serum Ig levels, as markers for immunity, are easy to measure. Most research on serum Ig measurement in IBD was done in the early 1960s and 1970s, prior to the current era of the wide use of anti-TNF biologics. No fixed pattern of Ig levels was noted in the studies, showing low, normal-to-elevated Ig levels [15–17, 41–44].

Our study showed concordant as well as discordant results when compared with earlier studies. Concordant results were the absence of a relationship between disease activity and Ig level in the majority of the patients and no difference in Ig levels between patients with various forms of IBD. A difference is noted in the prevalence of Ig deficiency when compared with old studies. In the majority of previous studies, IBD patients were found to have normal or elevated Ig levels. The Ig level was elevated transiently, corresponding to worse disease activity, which responded to therapy with corticosteroids or sulfasalazine. In healthy children, as well as in asymptomatic adults, selective IgA deficiency is the most common primary immune deficiency, with prevalence varying from 1 in 100 to 1 in 1000 [45–47]. In the setting of a normal IgG level in a healthy adult population, the prevalence of IgG subclass deficiency has been noted to be in the range of 2–20% [48–50]. In our study group, we found that approximately one-fifth of the patients had a low IgG1 or IgG, whereas a low IgM or IgA was less common. As our patients were not pooled from a recurrent sinupulmonary infection group, it is highly unlikely that high prevalence of Ig deficiency, especially IgG and IgG1, could be explained by characteristics of the study cohort.

Pathophysiology and treatment approaches for IBD are not completely understood. Current medical management aims to suppress the inflammation, but we have observed that there is no significant change in patients undergoing surgery. Keeping this in mind, the findings of our study have several clinical implications: none of the purported risk factors for Ig deficiency in the literature—such as the use corticosteroids, anti-TNF biologics, or immunomodulators, and abnormal liver or kidney function—were found to be associated with low Ig levels in multivariate analysis. So what could be possible reasons for this observed difference in the frequency of Ig deficiency? It could represent a subset of IBD patients having baseline innate immune deficiency. This may explain the therapeutic effect of IVIG or G-CSF, especially in some patients with medically refractory IBD. A recently published review article noted that immunoglobulin-containing protein preparations may offer a new strategy for restoring functional homeostasis in the intestinal tract of patients with enteropathy [51, 52]. Low Ig levels could also represent a yet-unknown complex pathophysiology of IBD, or be due to a paradigm shift, as compared with the past, in the overall medical and surgical approach towards patients. Also, with the increased lifespan of IBD patients, the disease duration of IBD has significantly increased. A low IgG or IgM was associated with increased age, whereas increase disease duration was associated with a low IgG1. Increased disease duration and age could be associated with protein-losing enteropathy, ultimately leading to low Ig levels.

There are limitations to our study. There might have been a referral bias, since all patients were from a tertiary care center. Most patients with IBD referred to our center had complex or refractory disease. However, the assessment of immune function, including the measurement of serum Ig, has become a part of our routine clinical practice. This is particularly true for one of our co-authors (B.S.) due to his practice pattern. As a result, approximately 30% of included patients had ileal pouches. However, we were able to obtain a sample size large enough for each category of disease studied. Another potential limitation of the study would be the cut-offs used in the study to define Ig deficiency. The number of patients with each diagnosis obliged us to divide them into quartiles, based on Ig levels, and to correlate disease activity or location with the low serum Igs. Another limitation of the study is its cross-sectional nature. We did not have baseline Ig levels and approximately 80% of the patients had their Ig levels measured only once, while the rest of them had Ig measured variable numbers of times.

In conclusion, our study showed that low Ig levels appear to be common in IBD patients. Increase in age, duration of IBD, hypoalbuminemia and the use of immunomodulators are risk factors. The findings of our study may shed some light on the disease course and areas for targeted therapy in IBD patients.

## Funding

This study was partially supported by a research grant from the Crohn’s and Colitis Foundation of America (to B. S.) and the Ed and Joey Story Endowed Chair (to B. S.)

*Conflict of interest statement:* none declared.

## Supplementary Material

Supplementary Data

## References

[1] Kirsner JB Historical aspects of inflammatory bowel disease. J Clin Gastroenterol 1988;10:286–97. 2980764 10.1097/00004836-198806000-00012

[2] Aronowitz R Spiro HM The rise and fall of the psychosomatic hypothesis in ulcerative colitis. J Clin Gastroenterol 1988;10:298–305. 2980765 10.1097/00004836-198806000-00013

[3] Duffy M O'Mahony L Coffey JC Sulfate-reducing bacteria colonize pouches formed for ulcerative colitis but not for familial adenomatous polyposis. Dis Colon Rectum 2002;45:384–8. 12068199 10.1007/s10350-004-6187-z

[4] Sullivan SN Hypothesis revisited: toothpaste and the cause of Crohn's disease. Lancet 1990;336:1096–7. 1977982 10.1016/0140-6736(90)92572-y

[5] Persson PG Ahlbom A Hellers G Diet and inflammatory bowel disease: a case-control study. Epidemiology 1992;3:47–52. 1313310 10.1097/00001648-199201000-00009

[6] Ramadas AV Gunesh S Thomas GA Natural history of Crohn's disease in a population-based cohort from Cardiff (1986-2003): a study of changes in medical treatment and surgical resection rates. Gut 2010;59:1200–6. 20650924 10.1136/gut.2009.202101

[7] Cannom RR Kaiser AM Ault GT Inflammatory bowel disease in the United States from 1998 to 2005: has infliximab affected surgical rates? Am Surg 2009;75:976–80. 19886148

[8] MacDermott RP Nash GS Auer IO Alterations in serum immunoglobulin G subclasses in patients with ulcerative colitis and Crohn's disease. Gastroenterology 1989;96:764–8. 2914639

[9] Muller-Ladner U Gross V Andus T Distinct patterns of immunoglobulin classes and IgG subclasses of autoantibodies in patients with inflammatory bowel disease. Eur J Gastroenterol Hepatol 1996;8:579–84. 8823574 10.1097/00042737-199606000-00016

[10] Gouni-Berthold I Baumeister B Berthold HK Immunoglobulins and IgG subclasses in patients with inflammatory bowel disease. Hepatogastroenterology 1999;46:1720–3. 10430330

[11] Savilahti E Sulphasalazine induced immunodeficiency. Br Med J (Clin Res Ed) 1983;287:759. 10.1136/bmj.287.6394.759 PMC1549039 6137259

[12] Klemola T Savilahti E Koskimies S Transient IgA and IgM deficiencies are frequent in children with ulcerative colitis. Eur J Pediatr 1988;147:184–7. 2896591 10.1007/BF00442219

[13] Soltis RD Wilson ID Serum immunoglobulin M concentrations following bowel resection in chronic inflammatory bowel disease. Gastroenterology 1975;69:885–92. 1100468

[14] Gelernt IM Present DH Janowitz HD Alterations in serum immunoglobulins after resection for ulcerative and granulomatous disease of the intestine. Gut 1972;13:21–3. 5060663 10.1136/gut.13.1.21 PMC1411977

[15] Marner IL Friborg S Simonsen E Disease activity and serum proteins in ulcerative colitis. Immunochemical quantitation. Scand J Gastroenterol 1975;10:537–44. 1153950

[16] Pepys MB Druguet M Klass HJ Immunological studies in inflammatory bowel disease. Ciba Found Symp 1977;46:283–304. 346325 10.1002/9780470720288.ch14

[17] Philipsen EK Bondesen S Andersen J Serum immunoglobulin G subclasses in patients with ulcerative colitis and Crohn's disease of different disease activities. Scand J Gastroenterol 1995;30:50–3. 7701250 10.3109/00365529509093235

[18] Rogosnitzky M Danks R Holt D Intravenous immunoglobulin for the treatment of Crohn's disease. Autoimmun Rev 2012;12:275–80. 22579561 10.1016/j.autrev.2012.04.006

[19] Rohr G Kusterer K Schille M Treatment of Crohn's disease and ulcerative colitis with 7S-immunoglobulin. Lancet 1987;1:170. 2880012 10.1016/s0140-6736(87)92013-7

[20] Chrissafidou A Malek M Musch E Experimental study on the use of intravenous immunoglobulin (IVIg) in patients with steroid-resistant Crohn's disease. Z Gastroenterol 2007;45:605–8. 17620224 10.1055/s-2007-963098

[21] Levine DS Fischer SH Christie DL Intravenous immunoglobulin therapy for active, extensive, and medically refractory idiopathic ulcerative or Crohn's colitis. Am J Gastroenterol 1992;87:91–100. 1728132

[22] Satsangi J Silverberg MS Vermeire S The Montreal classification of inflammatory bowel disease: controversies, consensus, and implications. Gut 2006;55:749–53. 16698746 10.1136/gut.2005.082909 PMC1856208

[23] Harvey RF Bradshaw JM A simple index of Crohn's-disease activity. Lancet 1980;1:514. 6102236 10.1016/s0140-6736(80)92767-1

[24] Schroeder KW Tremaine WJ Ilstrup DM Coated oral 5-aminosalicylic acid therapy for mildly to moderately active ulcerative colitis. A randomized study. N Engl J Med 1987;317:1625–9. 3317057 10.1056/NEJM198712243172603

[25] Gasche C Scholmerich J Brynskov J A simple classification of Crohn's disease: report of the Working Party for the World Congresses of Gastroenterology, Vienna 1998. Inflamm Bowel Dis 2000;6:8–15. 10701144 10.1097/00054725-200002000-00002

[26] Kawano T Matsuse H Obase Y Hypogammaglobulinemia in steroid-dependent asthmatics correlates with the daily dose of oral prednisolone. Int Arch Allergy Immunol 2002;128:240–3. 12119507 10.1159/000064258

[27] Settipane GA Pudupakkam RK McGowan JH Corticosteroid effect on immunoglobulins. J Allergy Clin Immunol 1978;62:162–6. 681628 10.1016/0091-6749(78)90101-x

[28] Cooper N Davies EG Thrasher AJ Repeated courses of rituximab for autoimmune cytopenias may precipitate profound hypogammaglobulinaemia requiring replacement intravenous immunoglobulin. Br J Haematol 2009;146:120–2. 19438506 10.1111/j.1365-2141.2009.07715.x

[29] Diwakar L Gorrie S Richter A Does rituximab aggravate pre-existing hypogammaglobulinaemia? J Clin Pathol 2010;63:275–7. 20203231 10.1136/jcp.2009.068940

[30] De La Torre I Leandro MJ Valor L Total serum immunoglobulin levels in patients with RA after multiple B-cell depletion cycles based on rituximab: relationship with B-cell kinetics. Rheumatology (Oxford) 2012;51:833–40. 22253030 10.1093/rheumatology/ker417

[31] Rackham OJ Sills JA Davidson JE Immunoglobulin levels in methotrexate treated paediatric rheumatology patients. Arch Dis Child 2002;87:147–8. 12138068 10.1136/adc.87.2.147 PMC1719204

[32] Levy J Barnett EV MacDonald NS The effect of azathioprine on gammaglobulin synthesis in man. J Clin Invest 1972;51:2233–8. 4629438 10.1172/JCI107031 PMC292386

[33] Guillemin F Bene MC Aussedat R Hypogammaglobulinemia associated with gold therapy: evidence for a partial maturation blockade of B cells. J Rheumatol 1987;14:1034–5. 3501467

[34] Williams A Scott DL Greenwood A The clinical value of measuring immunoglobulins when assessing penicillamine therapy in rheumatoid arthritis. Clin Rheumatol 1988;7:347–53. 3229080 10.1007/BF02239191

[35] Kemper MJ Altrogge H Ganschow R Serum levels of immunoglobulins and IgG subclasses in steroid sensitive nephrotic syndrome. Pediatr Nephrol 2002;17:413–17. 12107805 10.1007/s00467-001-0817-7

[36] Frasca D Landin AM Lechner SC Aging down-regulates the transcription factor E2A, activation-induced cytidine deaminase, and Ig class switch in human B cells. J Immunol 2008;180:5283–90. 18390709 10.4049/jimmunol.180.8.5283

[37] Lazuardi L Jenewein B Wolf AM Age-related loss of naive T cells and dysregulation of T-cell/B-cell interactions in human lymph nodes. Immunology 2005;114:37–43. 15606793 10.1111/j.1365-2567.2004.02006.x PMC1782064

[38] Segal AW Loewi G Neutrophil dysfunction in Crohn's disease. Lancet 1976;2:219–21. 59239 10.1016/s0140-6736(76)91024-2

[39] Korzenik JR Dieckgraefe BK An open-labelled study of granulocyte colony-stimulating factor in the treatment of active Crohn's disease. Aliment Pharmacol Ther 2005;21:391–400. 15709989 10.1111/j.1365-2036.2005.02287.x

[40] Dieckgraefe BK Korzenik JR Treatment of active Crohn's disease with recombinant human granulocyte-macrophage colony-stimulating factor. Lancet 2002;360:1478–80. 12433518 10.1016/S0140-6736(02)11437-1

[41] Weeke B Jarnum S Serum concentration of 19 serum proteins in Crohn's disease and ulcerative colitis. Gut 1971;12:297–302. 4102485 10.1136/gut.12.4.297 PMC1411620

[42] Armstrong-Esther CA Williams A Serum immunoglobulin levels in ulcerative colitis: an investigation. Nurs Times 1976;72:suppl:137–9. 972834

[43] Weeke B Bendixen G Serum immunoglobulins and organ-specific, cellular hypersensitivity in ulcerative colitis and Crohn's disease. Acta Med Scand 1969;186:87–91. 4185321 10.1111/j.0954-6820.1969.tb01444.x

[44] Bolton PM James SL Newcombe RG The immune competence of patients with inflammatory bowel disease. Gut 1974;15:213–19. 4841278 10.1136/gut.15.3.213 PMC1412882

[45] Carneiro-Sampaio MM Carbonare SB Rozentraub RB Frequency of selective IgA deficiency among Brazilian blood donors and healthy pregnant women. Allergol Immunopathol (Madr) 1989;17:213–16. 2816663

[46] Pereira LF Sapina AM Arroyo J Prevalence of selective IgA deficiency in Spain: more than we thought. Blood 1997;90:893. 9226194

[47] al-Attas RA Rahi AH Primary antibody deficiency in Arabs: first report from eastern Saudi Arabia. J Clin Immunol 1998;18:368–71. 9793829 10.1023/a:1023247117133

[48] Nahm MH Macke K Kwon OH Immunologic and clinical status of blood donors with subnormal levels of IgG2. J Allergy Clin Immunol 1990;85:769–77. 2324414 10.1016/0091-6749(90)90197-c

[49] Aucouturier P Mariault M Lacombe C Frequency of selective IgG subclass deficiency: a reappraisal. Clin Immunol Immunopathol 1992;63:289–91. 1623649 10.1016/0090-1229(92)90236-h

[50] Soderstrom T Soderstrom R Avanzini A Immunoglobulin G subclass deficiencies. Int Arch Allergy Appl Immunol 1987;82:476–80. 3570516 10.1159/000234258

[51] Petschow BW Blikslager AT Weaver EM Bovine immunoglobulin protein isolates for the nutritional management of enteropathy. World J Gastroenterol 2014;20:11713–26. 25206275 10.3748/wjg.v20.i33.11713 PMC4155361

[52] Petschow BW Burnett BP Shaw AL Dietary requirement for serum-derived bovine immunoglobulins in the clinical management of patients with enteropathy. Dig Dis Sci 2014 Aug 21. [Epub ahead of print] 10.1007/s10620-014-3322-0 PMC4284400 25142170

