# Supplementary material for: Frequency and risk factors of low immunoglobulin levels in patients with inflammatory bowel disease
Source: Gastroenterol Rep (Oxf). 2015 Jan 30;3(2):115–21. doi: 10.1093/gastro/gou082 (PMC4423463; doi:10.1093/gastro/gou082)
Supplement: Supplementary Data [file supp_3_2_115__index.html]

Frequency and risk factors of low immunoglobulin levels in patients with inflammatory bowel disease — Frequency and risk factors of low immunoglobulin levels in patients with inflammatory bowel disease — Supplementary Data 

# Frequency and risk factors of low immunoglobulin levels in patients with inflammatory bowel disease

## Supplementary Data

files

**Files in this Data Supplement:**

- Supplementary Data - docx file
