# Supplementary material for: Frequency and risk factors of low immunoglobulin levels in patients with inflammatory bowel disease
Source: Gastroenterol Rep (Oxf). 2015 Jan 30;3(2):115–21. doi: 10.1093/gastro/gou082 (PMC4423463; doi:10.1093/gastro/gou082)
Supplement: Supplementary Data [file supp_gou082_Supplement.docx]

**SUPPLEMENT**

**Table 1.** Univariate analysis of risk factors for low IgG level

| Characteristic | | All cases | Normal IgG level | IgG deficiency | *P* value |
| --- | --- | --- | --- | --- | --- |
| Number of patients | | 286 | 221 | 65 |  |
| Age at diagnosis of IBD, years | | 27.1±15.8 | 26.5±15.6 | 29.4±16.4 | 0.181 |
| Age at Ig test, years | | 38.7±17.9 | 36.9±17.7 | 44.9±17.3 | ***0.001*** |
| Duration from IBD to Ig test, years | | 7.6 (1.4-19.3) | 6.9 (0.9-17.6) | 14.7 (3.4-26.3) | ***0.004*** |
| Male gender, n (%) | | 141 | 104 (47.1%) | 37 (56.9%) | 0.162 |
| Caucasian patients, n (%) | | 237 | 178 (80.5%) | 59 (90.8%) | ***0.054*** |
| Body mass index, kg/m2 | | 25.4±6.1 | 25.3±6.2 | 25.9±5.7 | 0.517 |
| Smoking, n (%) | | | | | 0.162 |
| Never | | 187 | 150 (68.2%) | 37 (58.7%) |  |
| Ever | | 96 | 70 (31.8%) | 26 (41.3%) |  |
| Alcohol, n (%) | | | | | 0.423 |
| Never | | 185 | 146 (66.4%) | 39 (60.9%) |  |
| Ever | | 99 | 74 (33.6%) | 25 (39.1%) |  |
| Family history of IBD, n (%) | | 64 | 51 (23.1%) | 13 (20.6%) | 0.682 |
| Family history of colorectal cancer, n (%) | | 30 | 22 (10.0%) | 8 (12.7%) | 0.532 |
| History of IBD related bowel surgery, n (%) | | 188 | 139 (62.9%) | 49 (75.4%) | ***0.062*** |
| Ileal pouch construction, n (%) | | 65 | 56 (25.3%) | 9 (13.8%) | ***0.052*** |
| Type of IBD, n (%) | | | | | ***0.042*** |
| Crohn's disease | | 190 | 140 (63.3%) | 50 (76.9%) |  |
| UC or Indeterminate colitis | | 96 | 81 (36.7%) | 15 (23.1%) |  |
| Toxic or fulminant colitis, n (%) | | 24 | 19 (8.6%) | 5 (7.7%) | 0.817 |
| Fistulazing or stricturing CD phenotype, n (%) | | 113 | 76 (34.4%) | 37 (56.9%) | ***0.001*** |
| Primary sclerosing cholangitis, n (%) | | 20 | 19 (8.6%) | 1 (1.6%) | ***0.054*** |
| IBD-related hospitalization in last two years, n (%) | | 153 | 115 (54.5%) | 38 (58.5%) | 0.574 |
| Medications | | | | |  |
| 5-ASAs, n (%) | 91 | | 72 (32.7%) | 19 (29.2%) | 0.595 |
| Corticosteroids, n (%) | 108 | | 78 (35.5%) | 30 (46.2%) | 0.118 |
| 6-MP/azathioprine, n (%) | 50 | | 40 (18.2%) | 10 (15.6%) | 0.636 |
| Biologics, n (%) | 46 | | 39 (17.7%) | 7 (10.8%) | 0.180 |
| Methotrexate, n (%) | 15 | | 14 (6.4%) | 1 (1.5%) | 0.204 |
| Antibiotics, n (%) | 104 | | 78 (35.3%) | 26 (40.0%) | 0.488 |
| Laboratory tests | | | | |  |
| Anemia, n (%) | 178 | | 132 (60.3%) | 46 (70.8%) | 0.124 |
| Thrombocytosis, n (%) | 60 | | 49 (22.4%) | 11 (16.9%) | 0.344 |
| Leukocytosis, n (%) | 54 | | 41 (18.8%) | 13 (20.0%) | 0.830 |
| Hypoalbuminemia, n (%) | 97 | | 66 (30.4%) | 31 (48.4%) | ***0.008*** |
| Elevated alkaline phosphatase , n (%) | 46 | | 36 (16.7%) | 10 (15.6%) | 0.843 |
| Elevated aspartate aminotransferase, n (%) | 37 | | 30 (14.0%) | 7 (10.9%) | 0.524 |
| Elevated alanine aminotransferase, n (%) | 43 | | 36 (16.7%) | 7 (10.9%) | 0.264 |
| Increased total bilirubin, n (%) | 22 | | 18 (8.3%) | 4 (6.3%) | 0.792 |
| Hypocalcemia, n (%) | 61 | | 40 (18.3%) | 21 (33.9%) | ***0.009*** |
| Low Vitamin D, n (%) | 106 | | 75 (74.3%) | 31 (77.5%) | 0.688 |
| Low Ferritin, n (%) | 25 | | 17 (15.9%) | 8 (18.2%) | 0.730 |
| *C. difficile* positive, n (%) | 5 | | 5 (3.6%) | 0 (0.0%) | 0.324 |

**Table 2.** Univariate analysis of risk factors for low IgG1 level

| Characteristic | All cases | Normal IgG1 level | IgG1 deficiency | *P* value |
| --- | --- | --- | --- | --- |
| Number of patients | 124 | 95 | 29 |  |
| Age at diagnosis of IBD, years | 25.5±14.0 | 26.4±14.6 | 22.8±11.7 | 0.229 |
| Age at Ig test, years | 37.6±16.6 | 36.7±16.5 | 40.3±16.9 | 0.305 |
| Duration from IBD to Ig test, years | 9.5 (2.0-19.1) | 8.4 (1.7-15.1) | 18.3 (7.0-26.3) | ***0.011*** |
| Male gender, n (%) | 59 | 47 (49.5%) | 12 (41.4%) | 0.445 |
| Caucasian patients, n (%) | 105 | 80 (84.2%) | 25 (86.2%) | 1.0 |
| Body mass index, kg/m2 | 25.0±6.3 | 25.2±6.2 | 24.5±6.4 | 0.587 |
| Smoking, n (%) | | | | ***0.010*** |
| Never | 87 | 72 (76.6%) | 15 (51.7%) |  |
| Ever | 36 | 22 (23.4%) | 14 (48.3%) |  |
| Alcohol, n (%) | | | | 0.101 |
| Never | 87 | 70 (74.5%) | 17 (58.6%) |  |
| Ever | 36 | 24 (25.5%) | 12 (41.4%) |  |
| Family history of IBD, n (%) | 32 | 25 (26.3%) | 7 (24.1%) | 0.815 |
| Family history of colorectal cancer, n (%) | 15 | 9 (9.5%) | 6 (20.7%) | 0.189 |
| History of IBD related bowel surgery, n (%) | 98 | 73 (76.8%) | 25 (86.2%) | 0.278 |
| Ileal pouch construction, n (%) | 62 | 51 (53.7%) | 11 (37.9%) | 0.138 |
| Type of IBD, n (%) | |  |  | 0.118 |
| Crohn's disease | 57 | 40 (42.1%) | 17 (58.6%) |  |
| UC or Indeterminate colitis | 67 | 55 (57.9%) | 12 (41.4%) |  |
| Toxic or fulminant colitis, n (%) | 14 | 11 (11.6%) | 3 (10.3%) | 1.0 |
| Fistulazing or stricturing CD phenotype, n (%) | 31 | 24 (25.3%) | 7 (24.1%) | 0.903 |
| Primary sclerosing cholangitis, n (%) | 25 | 24 (25.3%) | 1 (3.4%) | ***0.010*** |
| IBD-related hospitalization in last two years, n (%) | 61 | 44 (47.8%) | 17 (63.0%) | 0.166 |
| Medications | | | | |
| 5-ASAs, n (%) | 35 | 29 (30.5%) | 6 (20.7%) | 0.303 |
| Corticosteroids, n (%) | 50 | 40 (42.1%) | 10 (34.5%) | 0.464 |
| 6-MP/azathioprine, n (%) | 19 | 16 (16.8%) | 3 (10.3%) | 0.559 |
| Biologics, n (%) | 24 | 21 (22.1%) | 3 (10.3%) | 0.161 |
| Methotrexate, n (%) | 4 | 3 (3.2%) | 1 (3.4%) | 1.0 |
| Antibiotics, n (%) | 64 | 49 (51.6%) | 15 (51.7%) | 0.989 |
| Laboratory tests | | | | |
| Anemia, n (%) | 74 | 49 (51.6%) | 25 (86.2%) | ***0.001*** |
| Thrombocytosis, n (%) | 32 | 26 (27.4%) | 6 (20.7%) | 0.472 |
| Leukocytosis, n (%) | 22 | 16 (16.8%) | 6 (20.7%) | 0.635 |
| Hypoalbuminemia, n (%) | 30 | 17 (18.3%) | 13 (44.8%) | ***0.004*** |
| Elevated alkaline phosphatase, n (%) | 26 | 19 (20.4%) | 7 (24.1%) | 0.670 |
| Elevated aspartate aminotransferase, n (%) | 21 | 17 (18.3%) | 4 (13.8%) | 0.779 |
| Elevated alanine aminotransferase, n (%) | 22 | 19 (20.4%) | 3 (10.3%) | 0.217 |
| Increased total bilirubin, n (%) | 10 | 8 (8.6%) | 2 (7.1%) | 1.0 |
| Hypocalcaemia, n (%) | 18 | 11 (11.7%) | 7 (25.9%) | 0.120 |
| Low vitamin d, n (%) | 52 | 40 (76.9%) | 12 (66.7%) | 0.532 |
| Low ferritin, n (%) | 14 | 10 (27.0%) | 4 (26.7%) | 1.0 |
| C. *difficile* positive, n (%) | 5 | 2 (2.6%) | 3 (11.5%) | 0.101 |

**Table 3.** Univariate analysis of risk factors for low IgA level

| Characteristic | All cases | Normal IgA level | IgA deficiency | *P* value |
| --- | --- | --- | --- | --- |
| Number of patients | 265 | 244 | 21 |  |
| Age at diagnosis of IBD, years | 27.3±15.4 | 27.3±15.2 | 27.9±18.7 | 0.868 |
| Age at Ig test, years | 38.8±18.2 | 38.9±18.0 | 36.8±21.1 | 0.612 |
| Duration from IBD to Ig test, years | 7.6 (1.3-19.3) | 7.9 (1.2-19.4) | 3.5 (1.3-15.4) | 0.372 |
| Male gender, n (%) | 127 | 115 (47.1%) | 12 (57.1%) | 0.378 |
| Caucasian patients, n (%) | 39 | 36 (14.8%) | 3 (14.3%) | 1.0 |
| Body mass index, kg/m2 | 25.4±6.0 | 25.5±6.0 | 23.4±5.3 | 0.115 |
| Smoking, n (%) |  |  |  | ***0.022*** |
| Never | 175 | 157 (64.9%) | 18 (90.0%) |  |
| Ever | 87 | 85 (35.1%) | 2 (10.0%) |  |
| Alcohol, n (%) |  |  |  | ***0.056*** |
| Never | 176 | 158 (65.3%) | 18 (85.7%) |  |
| Ever | 87 | 84 (34.7%) | 3 (14.3%) |  |
| Family history of IBD, n (%) | 55 | 49 (20.2%) | 6 (28.6%) | 0.402 |
| Family history of colorectal cancer, n (%) | 28 | 24 (9.9%) | 4 (19.7%) | 0.256 |
| History of IBD related bowel surgery, n (%) | 180 | 167 (68.4%) | 13 (61.9%) | 0.538 |
| Ileal pouch construction, n (%) | 76 | 71 (29.1%) | 5 (23.8%) | 0.607 |
| Type of IBD, n (%) | | | | 0.752 |
| Crohn's disease | 160 | 148(60.7%) | 12 (57.1%) |  |
| UC or indeterminate colitis | 105 | 96 (39.3%) | 9 (42.9%) |  |
| Toxic or fulminant colitis, n (%) | 22 | 22 (9.0%) | 0 (0%) | 0.233 |
| Fistulazing or stricturing CD phenotype, n (%) | 94 | 90 (36.9%) | 4 (19.0%) | 0.101 |
| Primary sclersoing chloangitis, n (%) | 25 | 24 (9.9%) | 1 (4.8%) | 0.704 |
| IBD-related hospitalization in last two years, n (%) | 138 | 129 (55.4%) | 9 (42.9%) | 0.270 |
| Medications | | | | |
| 5-ASAs, n (%) | 84 | 76 (31.3%) | 8 (38.1%) | 0.520 |
| Corticosteroids, n (%) | 102 | 94 (38.7%) | 8 (38.1%) | 0.958 |
| 6-MP/azathioprine, n (%) | 44 | 37 (15.2%) | 7 (33.3%) | ***0.035*** |
| Biologics, n (%) | 41 | 38 (15.6%) | 3 (14.3%) | 1.0 |
| Methotrexate, n (%) | 13 | 11 (4.5%) | 2 (9.5%) | 0.608 |
| Antibiotics, n (%) | 103 | 96 (39.3%) | 7 (33.3%) | 0.588 |
| Laboratory tests | | | | |
| Anemia, n (%) | 163 | 150 (62.0%) | 13 (61.9%) | 0.994 |
| Thrombocytosis, n (%) | 61 | 52 (21.5%) | 9 (42.9%) | ***0.033*** |
| Leukocytosis, n (%) | 45 | 43 (17.8%) | 2 (9.5%) | 0.392 |
| Hypoalbuminemia, n (%) | 87 | 81 (33.9%) | 6 (28.6%) | 0.620 |
| Elevated alkalaine phosphatase, n (%) | 44 | 42 (17.6%) | 2 (9.5%) | 0.394 |
| Elevated aspartate aminotransferase, n (%) | 32 | 30 (12.7%) | 2 (9.5%) | 0.755 |
| Elevated alanine aminotransferase, n (%) | 37 | 35 (14.7%) | 2 (9.5%) | 0.748 |
| High total bilirubin, n (%) | 22 | 18 (7.6%) | 4 (19.0%) | ***0.089*** |
| Hypocalcemia, n (%) | 54 | 51 (21.4%) | 3 (14.3%) | 0.581 |
| Low vitamin D, n (%) | 108 | 101 (76.5%) | 7 (58.3%) | 0.295 |
| Low ferritin, n (%) | 23 | 20 (16.0%) | 3 (25.0%) | 0.690 |
| C. *difficile* positive, n (%) | 6 | 6 (3.6%) | 0 (0%) | 0.662 |

**Table 4.** Univariate analysis of risk factors for low IgM level

| Characteristic | All cases | Normal IgM level | IgM deficiency | P value |
| --- | --- | --- | --- | --- |
| Number of patients | 220 | 196 | 24 |  |
| Age at diagnosis of IBD, years | 26.9±14.3 | 25.7±14.1 | 36.1±13.3 | ***0.001*** |
| Age at Ig test, years | 38.4±18.0 | 36.5±17.5 | 53.4±14.3 | ***<0.001*** |
| Duration from IBD to Ig test, years | 7.6 (1.5-19.5) | 6.8 (1.0-17.8) | 19.3 (8.7-26.1) | ***0.006*** |
| Male gender, n (%) | 105 | 92 (46.9%) | 13 (54.2%) | 0.503 |
| Caucasian patients, n (%) | 185 | 164 (83.7%) | 21 (87.5%) | 0.774 |
| Body mass index, kg/m2 | 25.6±6.2 | 25.3±5.7 | 28.1±9.0 | ***0.037*** |
| Smoking, n (%) | | | | 0.671 |
| Never | 144 | 129 (66.8%) | 15 (62.5%) |  |
| Ever | 73 | 64 (33.2%) | 9 (37.5%) |  |
| Alcohol, n (%) | | | | 0.659 |
| Never | 145 | 130 (67.0%) | 15 (62.5%) |  |
| Ever | 73 | 64 (33.0%) | 9 (37.5%) |  |
| Family history of IBD, n (%) | 48 | 47 (24.2%) | 1 (4.2%) | ***0.025*** |
| Family history of colorectal cancer, n (%) | 22 | 20 (10.3%) | 2 (8.3%) | 1.0 |
| History of IBD related bowel surgery, n (%) | 147 | 128 (65.3%) | 19 (79.2%) | 0.173 |
| Ileal pouch construction, n (%) | 53 | 50 (25.5%) | 3 (12.5%) | 0.159 |
| Type of IBD, n (%) | | | | 0.123 |
| Crohn's disease | 143 | 124 (63.3%) | 19 (79.2%) |  |
| UC or Indeterminate colitis | 77 | 72 (36.7%) | 5 (20.8%) |  |
| Ileal pouch construction, n (%) | 53 | 50 (25.5%) | 3 (12.5%) | 0.159 |
| Toxic or fulminant colitis, n (%) | 19 | 18 (9.2%) | 1 (4.2%) | 0.495 |
| Fistulazing or stricturing CD phenotype, n (%) | 86 | 76 (38.8%) | 10 (41.7%) | 0.784 |
| Primary sclerosing cholangitis, n (%) | 13 | 11 (5.7%) | 2 (8.3%) | 0.641 |
| IBD-related hospitalization in last two years, n (%) | 116 | 104 (55.9%) | 12 (50.0%) | 0.583 |
| Medications | | | | |
| 5-ASAs, n (%) | 73 | 63 (32.3%) | 10 (41.7%) | 0.359 |
| Corticosteroids, n (%) | 86 | 71 (36.4%) | 15 (62.5%) | ***0.014*** |
| 6-MP/azathioprine, n (%) | 38 | 34 (17.4%) | 4 (16.7%) | 1.0 |
| Biologics, n (%) | 38 | 34 (17.4%) | 4 (16.7%) | 1.0 |
| Methotrexate, n (%) | 12 | 11 (5.6%) | 1 (4.2%) | 1.0 |
| Antibiotics, n (%) | 80 | 72 (36.7%) | 8 (33.3%) | 0.734 |
| Laboratory tests | | | | |
| Anemia, n (%) | 137 | 119 (61.3%) | 18 (75.0%) | 0.191 |
| Thrombocytosis, n (%) | 50 | 48 (24.7%) | 2 (8.3%) | ***0.071*** |
| Leukocytosis, n (%) | 39 | 35 (18.1%) | 4 (16.7%) | 1.0 |
| Hypoalbuminemia, n (%) | 73 | 59 (30.7%) | 14 (60.9%) | ***0.004*** |
| Elevated alkaline phosphatase, n (%) | 33 | 31 (16.2%) | 2 (8.7%) | 0.393 |
| Elevated aspartate aminotransferase, n (%) | 23 | 20 (10.6%) | 3 (13.0%) | 0.722 |
| Elevated alanine aminotransferase, n (%) | 28 | 26 (13.6%) | 2 (8.7%) | 0.556 |
| High total bilirubin, n (%) | 12 | 10 (5.2%) | 2 (8.7%) | 0.623 |
| Hypocalcemia, n (%) | 43 | 33 (17.2%) | 10 (43.5%) | ***0.006*** |
| Low vitamin D, n (%) | 88 | 78 (75.0%) | 10 (76.9%) | 1.0 |
| Low ferritin, n (%) | 19 | 17 (16.8%) | 2 (12.5%) | 0.741 |
| *C. difficile* positive, n (%) | 4 | 4 (3.2%) | 0 (0.0%) | 1.0 |
